# Supplementary material for: The Safety of mRNA-1273, BNT162b2 and JNJ-78436735 COVID-19 Vaccines: Safety Monitoring for Adverse Events Using Real-World Data
Source: Vaccines (Basel). 2022 Feb 17;10(2):320. doi: 10.3390/vaccines10020320 (PMC8879025; doi:10.3390/vaccines10020320)
Supplement: Supplementary file 1 [file vaccines-10-00320-s001.zip › vaccines-1539807-supplementary.pdf]

Supplementary Materials:

The Safety of mRNA-1273, BNT162b2, and JNJ-78436735 COVID-19 Vaccines: Safety Monitoring for Adverse Events Using Real-World Data

Soonok Sa, Chae Won Lee, Sung Ryul Shim, Hyounggyoon Yoo, Jinwha Choi, Ju Hee Kim, Kiwon Lee, Myunghee Hong* and Hyun Wook Han*

This supplementary material contains **additional one figure**, and **six tables**.

Table S1. Characteristics of individuals who report at least one adverse event after COVID-19 vaccination

	mRNA-1273	BNT162b2	JNJ-78436735	**Sum**
**Females**				
18-24	8,153	9,291	2,312	19,756
25-39	39,654	39,934	6,990	86,578
40-49	29,968	29,643	4,929	64,540
50-64	47,049	40,449	7,373	94,871
65-74	32,345	17,672	1,927	51,944
≥75	18,534	9,978	791	29,303
**Sum**	175,703	146,967	24,322	346,992
**Males**				
18-24	3,717	4,820	2,017	10,554
25-39	11,992	14,364	4,303	30,659
40-49	8,303	9,437	2,372	20,112
50-64	15,838	15,117	3,934	34,889
65-74	12,634	8,816	1,111	22,561
≥75	8,971	5,915	519	15,405
**Sum**	61,455	58,469	14,256	134,180
**SUM of VAERES**	**237,158 (0.15656%)**	**205,436 (0.09211%)**	**38,578 (0.25718%)**	**481,172 (0.12353%)**

Table S2. Classification of severe AEs

Severe AEs	Groups
Bell's palsy<br>stroke, ischemia<br>stroke, hemorrhagic<br>encephalitis/myelitis/encephalomyelitis<br>cerebral venous sinus thrombosis<br>convulsion/seizures<br>Guillain–Barré syndrome<br>transverse myelitis<br>acute disseminated encephalomyelitis<br>narcolepsy/cataplexy	central nervous disorders
pulmonary embolism<br>acute respiratory distress syndrome (ARDS)	respiratory disorders
acute myocardial infarction<br>myocarditis/pericarditis	cardiac disorders
appendicitis	gastrointestinal disorders
anemia<br>lymphadenopathy<br>lymphopenia<br>neutropenia<br>other thrombosis<br>thrombocytopenia<br>deep vein thrombosis	hematologic disorders
anaphylaxis<br>multisystem inflammatory syndrome in children/adults<br>death	others

Table S3. Diagnostic codes used to identify AEs and onset date.

**Severe AEs for Comparative Analyses**	**MedDRA code(s),** ver. 24.0
Bell's palsy	10004223, 10030069
Stroke, hemorrhagic	10042316, 10073564, 10019016, 10055677
Stroke, ischemic	10044390, 10060840, 10061256, 10043647
Encephalitis / myelitis / encephalomyelitis	10014581, 10014589, 10014619, 10058994, 10076948, 10082097, 10074713, 10048999
Cerebral venous sinus thrombosis	10083037
Convulsions / seizures	10003628, 10056699, 10049612, 10053398, 10010145, 10052391, 10010904, 10010920, 10015037,10077380, 10016284, 10018090, 10083376, 10018100, 10018659, 10071081, 10054859, 10056209, 10061334, 10034759, 10039906, 10039907, 10071350, 10040703, 10041962, 10043209, 10051171, 10043994
Guillain-Barré syndrome	10018767, 10049567, 10061811
Transverse myelitis	10028527
Acute disseminated encephalomyelitis	10000709
Narcolepsy / cataplexy	10007737, 10028713
Pulmonary embolism	10037377, 10063909, 10069909, 10083093
Acute respiratory distress syndrome	10001052
Acute myocardial infarction	10000891
Myocarditis / pericarditis	10064539, 10079058, 10014961, 10081004, 10082606, 10028606, 10034484, 10034486, 10034487, 10059361, 10051071
Appendicitis	10003011, 10063063, 10003012, 10081534
Anemia	10022972, 10061101, 10002080, 10002081, 10037006, 10018916, 10002967
Lymphadenopathy	10025197
Lymphopenia	10025327
Neutropenia	10029354, 10059482
Other thrombosis	10014513, 10014522, 10048632, 10036206, 10006537, 10003880, 10047249, 10002910, 10003192,10007688, 10007830, 10008023, 10008092, 10008132, 10008138, 10019713, 10023237, 10027397, 10027402, 10034272, 10034324, 10036300, 10037340, 10037437, 10037459, 10038548, 10038908, 10041659, 10049446, 10082030, 10074349, 10072059, 10038547, 10078810, 10081850
Thrombocytopenia	10043554, 10058423, 10076744, 10076747, 10078387, 10083842
Deep vein thrombosis	10051055, 10043570, 10043581, 10043595
Anaphylaxis	10002198, 10002199, 10002216, 10063119
Multisystem inflammatory syndrome in children/adults	10084767, 10051379
Death	10011906

Table S4. Characteristics of serious outcomes for the three vaccines

	mRNA-1273	BNT162b2	JNJ-78436735
Hospital Days (median)	7,997 (3)	9,246 (3)	2,306 (3)
Disable (%)	2,803 (0.00185%)	3,696 (0.001635%)	851 (0.005673%)
ER visit (%)	20 (0%)	27 (0%)	1 (0%)
Died (%)	2,718 (0.001794%)	2,428 (0.001074%)	546 (0.0036399%)
**Total**	**13,538 (0.0089371%)**	**15,397 (0.0068118%)**	**3,704 (0.024693%)**

Table S5. Multivariate regression analysis for major AEs by sex (coding male as 1 and female as 0), age (years), onset days (number of days), and vaccine type (coding mRNA vaccines as 0 and viral vector vaccine as 1) as covariates. Dependent variables: incidence, independent variables: sex (coding male as 1 and female as 0), age (years), onset days (number of days), and vaccine type.

	Death			Anaphylaxis			Overall severe AEs		
	OR	CI	*p*	OR	CI	*p*	OR	CI	*p*
Sex (M/F)	2.761	2.599 – 2.933	< 0.001	0.532	0.465 – 0.608	< 0.001	1.374	1.342 – 1.406	< 0.001
Age (years)	1.086	1.084 – 1.089	< 0.001	0.989	0.986 – 0.992	< 0.001	1.001	1.001 – 1.002	< 0.001
Onset (days)	1.007	1.007 – 1.008	< 0.001	0.959	0.949 – 0.969	< 0.001	1.005	1.005 – 1.006	< 0.001
Vaccine types*	1.901	1.713 – 2.111	< 0.001	0.619	0.495 – 0.772	< 0.001	1.044	1.005 – 1.086	0.028

	Bell's palsy			Guillain–Barré syndrome			Cerebral venous sinus thrombosis		
	OR	CI	*p*	OR	CI	*p*	OR	CI	*p*
Sex (M/F)	2.042	1.885 – 2.213	< 0.001	2.310	1.931 – 2.764	< 0.001	0.942	0.648 – 1.369	0.755
Age (years)	1.003	1.000 – 1.005	0.025	1.015	1.010– 1.020	< 0.001	0.991	0.981 – 1.001	0.072
Onset (days)	1.008	1.007 – 1.009	< 0.001	1.008	1.006 – 1.010	< 0.001	1.013	1.009 – 1.016	< 0.001
Vaccine types*	0.923	0.798 – 1.067	0.279	4.183	3.411 – 5.130	< 0.001	5.049	3.492 – 7.301	< 0.001

	Myocarditis/Pericarditis			Acute myocardial infarction			Acute respiratory distress syndrome		
	OR	CI	*p*	OR	CI	*p*	OR	CI	*p*
Sex (M/F)	5.614	5.074 – 6.211	< 0.001	3.593	2.994 – 4.312	< 0.001	2.167	1.430 – 3.283	< 0.001
Age (years)	0.955	0.952 – 0.958	< 0.001	1.037	1.032 – 1.043	< 0.001	1.050	1.036 – 1.064	< 0.001
Onset (days)	1.009	1.008 – 1.011	< 0.001	1.007	1.005 – 1.009	< 0.001	1.015	1.012 – 1.018	< 0.001
Vaccine types*	0.378	0.305 – 0.469	< 0.001	1.130	0.818 – 1.562	0.458	2.951	1.706 – 5.105	< 0.001

	Pulmonary embolism			Thrombocytopenia			Deep vein thrombosis		
	OR	CI	*p*	OR	CI	*p*	OR	CI	*p*
Sex (M/F)	2.002	1.832 – 2.188	< 0.001	1.827	1.550 – 2.154	< 0.001	2.103	1.910 – 2.315	< 0.001
Age (years)	1.023	1.020 – 1.025	< 0.001	1.022	1.017 – 1.027	< 0.001	1.022	1.019 – 1.025	< 0.001
Onset (days)	1.009	1.008 – 1.010	< 0.001	1.009	1.007 – 1.011	< 0.001	1.008	1.007 – 1.009	< 0.001
Vaccine types*	3.108	2.782 – 3.472	< 0.001	2.421	1.943 – 3.016	< 0.001	4.015	3.588 – 4.493	< 0.001

	Lymphadenopathy			Overall common AEs			COVID - 19		
	OR	CI	*p*	OR	CI	*p*	OR	CI	*p*
Sex (M/F)	0.524	0.501 – 0.548	< 0.001	0.621	0.612 – 0.630	< 0.001	1.527	1.463 – 1.593	< 0.001
Age (years)	0.984	0.983 – 0.985	< 0.001	0.999	0.999 – 0.999	< 0.001	1.010	1.009 – 1.012	< 0.001
Onset (days)	0.993	0.992 – 0.994	< 0.001	1.000	1.000 – 1.001	0.030	1.031	1.031 – 1.031	< 0.001
Vaccine types*	0.314	0.284 – 0.346	< 0.001	1.314	1.280 – 1.350	< 0.001	1.396	1.307 – 1.490	< 0.001

*Two mRNA vaccines/one viral vector vaccine

OR: Odds Ratio, CI: 95% confidence intervals, *p*: *p*-values

Table S6. Multivariate regression analysis for major AEs by sex (coding male as 1 and female as 0), age (years), onset of symptoms (number of days), and vaccine types (reference: BNT162b2) as covariates. Dependent variables: incidence, independent variables: sex (coding male as 1 and female as 0), age (years), onset of symptoms (days), and vaccine type.

	Death (N = 4,589)	AP (N = 1,610)	Overall severe AEs (N = 36,077)	BP (N = 2,505)	GBS (N = 510)	CVST (N = 133)	MP (N = 1,766)	AMI (N = 494)	ARDS (N = 92)	PE (N = 2,046)	TB (N = 599)	DVT (N=1,733)	LP (N = 15,018)	Overall common AEs (N = 369,788)	COVID-19 (N=13,509)	DRD[†] (N=6,183)
Sex (M/F)	2.748*** (2.586 – 2.919)	0.525*** (0.460 – 0.600)	1.363*** (1.332 – 1.395)	2.028*** (1.871 – 2.197)	2.290*** (1.914 – 2.741)	0.941 (0.647 – 1.367)	5.575*** (5.039 – 6.169)	3.552*** (2.960 – 4.264)	2.161*** (1.426 – 3.274)	1.995*** (1.826 – 2.180)	1.812*** (1.537 – 2.137)	2.088*** (1.897 – 2.300)	0.519*** (0.497 – 0.543)	0.627*** (0.618 – 0.636)	1.502*** (1.439 – 1.568)	–
Age (years)	1.087*** (1.084 – 1.089)	0.990*** (0.987 – 0.993)	1.002*** (1.001 – 1.003)	1.003** (1.001 – 1.006)	1.016*** (1.011 – 1.021)	0.991 (0.981 – 1.001)	0.956*** (0.953 – 0.959)	1.038*** (1.033 – 1.044)	1.050*** (1.036 – 1.064)	1.023*** (1.020 – 1.026)	1.812*** (1.537 – 2.137)	1.022*** (1.020 – 1.025)	0.985*** (0.984 – 0.986)	0.998*** (0.997 – 0.998)	1.012*** (1.011 – 1.013)	0.949*** (0.947 – 0.951)
Onset (days)	1.007*** (1.006 – 1.008)	0.962*** (0.952 – 0.971)	1.002*** (1.001 – 1.003)	1.008*** (1.007 – 1.009)	1.008*** (1.006 – 1.010)	1.012*** (1.009 – 1.016)	1.009*** (1.008 – 1.010)	1.007*** (1.005 – 1.009)	1.015*** (1.012 – 1.018)	1.009*** (1.008 – 1.010)	1.009*** (1.007 – 1.010)	1.007*** (1.006 – 1.009)	0.993*** (0.992 – 0.994)	1.001*** (1.000 – 1.001)	1.031*** (1.03 – 1.031)	1.008*** (1.007 – 1.009)
Vaccine type (JNJ-78436735)	1.739*** (1.559 – 1.941)	0.513*** (0.409 – 0.643)	0.928*** (0.891 – 0.966)	0.833* (0.717 – 0.968)	3.565*** (2.852 – 4.457)	4.829*** (3.175 – 7.343)	0.346*** (0.278 – 0.431)	0.964 (0.691 – 1.346)	2.767*** (1.532 – 4.997)	2.916*** (2.584 – 3.290)	2.112*** (1.671 – 2.669)	3.536*** (3.126 – 4.001)	0.280*** (0.254 – 0.310)	1.550*** (1.508 – 1.593)	1.083* (1.012 – 1.158)	0.992 (0.909 – 1.083)
Vaccine type (mRNA-1273)	0.848*** (0.796 – 0.904)	0.663*** (0.597 – 0.735)	0.784*** (0.766 – 0.802)	0.806*** (0.742 – 0.877)	0.714** (0.578 – 0.882)	0.913 (0.601 – 1.386)	0.816*** (0.739 – 0.901)	0.720*** (0.598 – 0.868)	0.879 (0.557 – 1.388)	0.881* (0.797 – 0.973)	0.755** (0.630 – 0.903)	0.772*** (0.690 – 0.863)	0.792*** (0.765 – 0.819)	1.400*** (1.380 – 1.421)	0.566*** (0.542 – 0.592)	0.593*** (0.561 – 0.628)

The odds ratio was calculated by multivariate logistic regression analysis for each severe AEs after adjusting for sex (reference: female), age, onset days of symptoms (number of days), and vaccine type (reference: BNT162b2) as covariates. AMI, Acute myocardial infarction; AP, Anaphylaxis; ARDS, Acute respiratory distress syndrome; BP, Bell's palsy; CVST, cerebral venous sinus thrombosis; DRD, Dysmenorrhea and related disorders (Dysmenorrhea or disruption of menstrual cycles); DVT, deep vein thrombosis; GBS, Guillain–Barré syndrome; LP, lymphadenopathy; MP, Myocarditis/Pericarditis; PE, pulmonary embolism; TB, thrombocytopenia.

p<0.05*, ** *p<0.01*, * *p<0.001.*

[†]This analysis was conducted only on women.

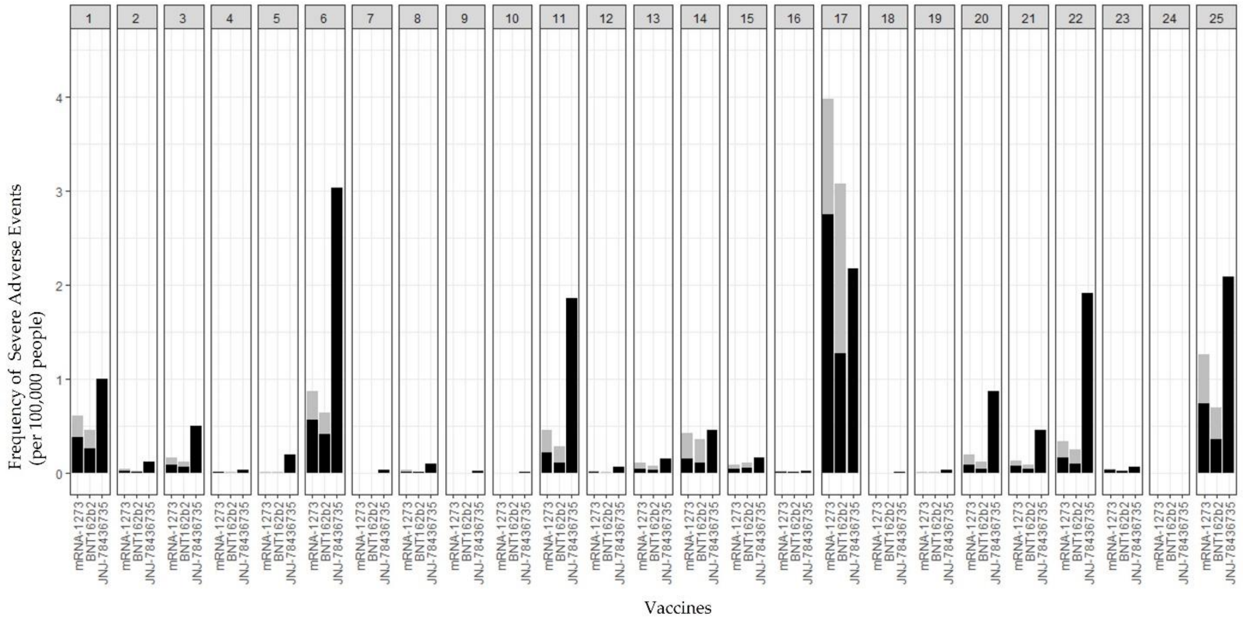
Frequency of Severe Adverse Events (per 100,000 people)
Vaccines
mRNA-1273
BNT162b2
JNJ-78436735

(a)

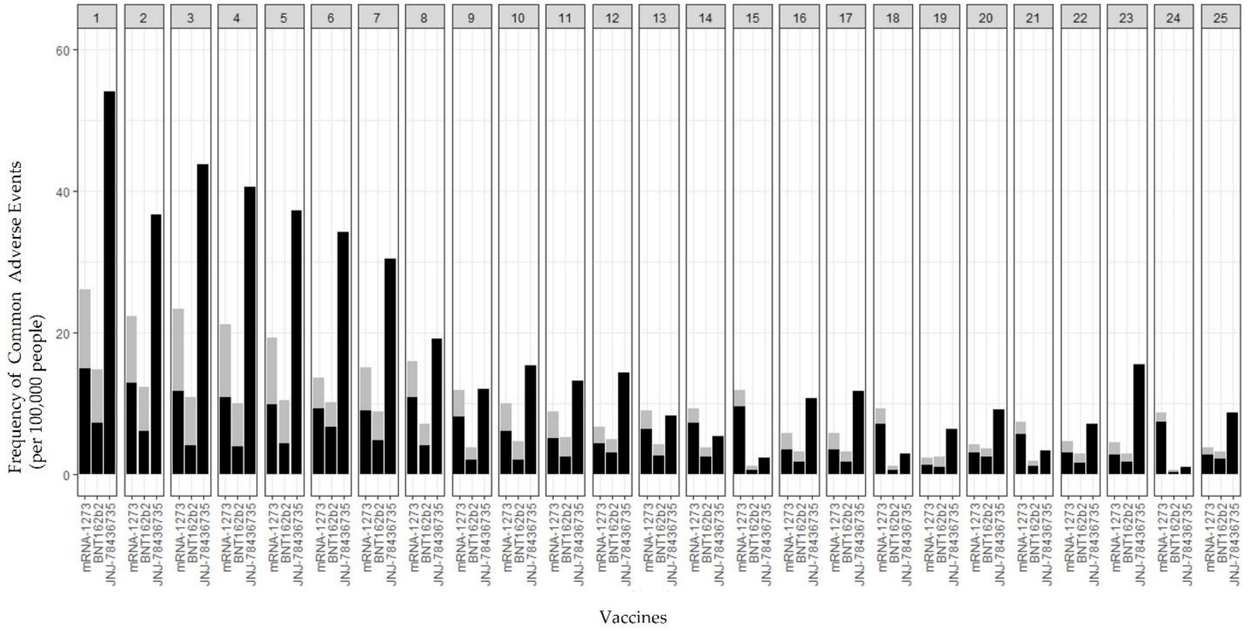
Frequency of Common Adverse Events (per 100,000 people)
Vaccines
mRNA-1273
BNT162b2
JNJ-78436735

(b)

Figure S1. Incidence (per 100,000 people) of specific AEs among recipients of either of the two mRNA vaccines (mRNA-1273, and BNT162b2) or the one viral vector vaccine (JNJ-78436735). Comparative incidence per 100,000 people of each 25 AEs among the three types of vaccines : (a) Incidence of severe AEs of the 25 groups after vaccination (1:Bell's palsy; 2:Stroke, hemorrhagic; 3:Stroke, ischemic; 4:Encephalitis/myelitis/encephalomyelitis; 5:Cerebral venous sinus thrombosis; 6:Convulsions/seizures; 7: Guillain–Barré syndrome; 8:Transverse myelitis; 9:Acute disseminated encephalomyelitis; 10:Narcolepsy/cataplexy; 11:Pulmonary embolism; 12:Acute respiratory distress syndrome; 13:Acute myocardial infarction; 14:Myocarditis/pericarditis; 15:Appendicitis; 16:Anemia; 17:Lymphadenopathy; 18:Lymphopenia; 19:Neutropenia; 20:Other thrombosis; 21:Thrombocytopenia; 22:Deep vein thrombosis; 23:Anaphylaxis; 24:Multisystem inflammatory syndrome in children/adults, 25:Death); (b) Incidence of the 25 common AEs after vaccination (1:Headache, 2:Fatigue, 3:Pyrexia, 4:Chills, 5:Pain, 6:Dizziness, 7:Nausea, 8:Pain in extremity, 9:Injection site pain, 10:Myalgia, 11:Arthralgia, 12:Dyspnoea, 13:Rash, 14:Pruritus, 15:Injection site erythema, 16:Asthenia, 17:Vomiting, 18:Injection site swelling, 19:COVID-19, 20:Paraesthesia, 21:Erythema, 22:Diarrhoea, 23:Hyperhidrosis, 24:Injection site pruritus, 25: Hypoesthesia) ; (b) Incidence of severe AEs of the 25 groups after vaccination (The ranked top-25 common AEs were listed in order of incidence) , dose 1(the first dose): black , dose 2 (the second dose): grey.

+ This graph consists of data in which doses are recorded as 1 or 2.
